# An animated educational video is effective for improving treatment outcomes in pediatric patients with atopic dermatitis: A prospective observational trial

**DOI:** 10.1016/j.jdin.2026.02.001

**Published:** 2026-02-13

**Authors:** Eve Finkelstein, Ayelet Ollech, Irene Unterman, Michal Neumark, Vered Molho-Pessach

**Affiliations:** a Department of Dermatology, Faculty of Medicine, Hebrew University of Jerusalem, Pediatric Dermatology Service, Hadassah Medical Center, Jerusalem, Israel; b Faculty of Medicine, Pediatric Dermatology Service, Shaare Zedek Medical Center, Hebrew University of Jerusalem, Jerusalem, Israel

**Keywords:** animation, atopic dermatitis, educational video, patient education, pediatric

*To the Editor:* Effective pediatric atopic dermatitis (AD) care requires families to understand the treatment plan.^1^ Clinicians face time constraints that limit comprehensive patient education,^2^ underscoring the need for supplementary educational resources.^3^

This study evaluates whether a 7-minute-long animated educational video enhances disease outcomes in pediatric AD. A script was written by pediatric dermatologists at Hadassah Medical Center and then animated by Silueta Productions (Tel Aviv).

We performed a prospective observational frequency-matched case-control trial involving children with AD aged 2 months to 18 years at 2 tertiary medical centers in Israel, following approval by local institutional review boards.

AD diagnosis was confirmed by certified dermatologists using the American Academy of Dermatology diagnostic criteria.^4^ Children attending their first visit for AD were included. We excluded patients receiving systemic treatment (methotrexate, cyclosporine, azathioprine, mycophenolate mofetil, dupilumab, upadacitinib, and systemic corticosteroids).

Outcome measures were disease severity (eczema area and severity index [EASI] and scoring atopic dermatitis [SCORAD]), pruritus (peak pruritus numerical rating scale), and quality of life (children dermatology life quality index [CDLQI]).

Participants were matched into 2 groups by age, gender, and disease severity scores and were given an oral and written treatment plan based on the Consensus-based European guidelines for treatment of AD.^5^ The study group was emailed a link to the video and was encouraged to watch it weekly for the first month. Parents were required to watch the video; if the child was old enough, he was encouraged to watch it as well.

Participants were followed up in clinic or via teledermatology at 1 month and 3 months, and SCORAD, EASI, pruritus numerical rating scale, and CDLQI were assessed.

Descriptive statistics were used to display results. Categorical variables were compared by the χ^2^ test, and continuous variables by Student *t* test or ANOVA for repeated measures. Analysis was performed using SPSS (version 25.0).

Fifty children were recruited in each group. Seventy-five patients attended at least 1 follow-up visit, 40 in the video group and 35 in the control group. Demographics are presented in Table I. At the first visit, there were no statistically significant differences between the groups (Table II), although all scores tended to be higher in the video group.

**Table I:** Demographics and baseline clinical characteristics of study participants

Variable group	Video	Control	*P* value
Gender (male), M (%)	30 (73)	22 (54)	.109
Age, M (SD)	39.9 (44.4)	49.9 (47.8)	.326

**Table II:** Disease severity scores

Variable group	Video		Control		Effect size	*P* value
	V1	V3	V1	V3		
EASI∗	15.4 (12.5)	2.3 (2.8)	10.5 (9.6)	4.0 (8.3)	.089	.008
SCORAD∗	48.3 (19.7)	13.3 (11.7)	42.1 (18.0)	21.0 (15.8)	.116	.002
CDQLI∗	10.5 (5.6)	3.0 (4.2)	9.0 (5.8)	4.7 (4.3)	.094	.007
pNRS∗	6.6 (2.9)	2.3 (2.9)	5.8 (2.8)	3.2 (2.9)	.062	.030

*pNRS*, Pruritus numerical rating scale; *V1*, visit 1; *V3*, visit 3.

∗ Two-way ANOVA for repeated measures. Effect size–Partial Eta squared.

In both groups, EASI, SCORAD, and CDLQI scores improved significantly over time, improvement was significantly greater in the video group compared to the control group at the third visit versus baseline (Table II, Supplementary Fig 1, available via Mendeley at https://doi.org/10.17632/zbkj9pg26y.1). Pruritus improved significantly better in the video group compared to the control group only between the first and second visits (*P* value = .022).

Our animated video is a valuable tool for improving AD severity and quality of life over the course of 3 months in a pediatric AD population.

It is available on YouTube in English, Hebrew, and Arabic (QR codes are available below).

A limitation of our study is that the study group received the video by email. Some parents used the email to request extra guidance, which may have affected compliance.

The video could be a time-saving tool for physicians treating children with AD and can serve as an aid in improving AD outcomes.

The video can be accessed here:

In English: https://youtu.be/N1mfeETSNkQ.

In Hebrew: https://youtu.be/NcEXgEkBSZI.

In Arabic: https://youtu.be/AKSllcbMm8M.

### Declaration of generative AI and AI-assisted technologies in the writing process

During the preparation of this work, the author(s) used [ChatGPT] in order to adjust the style and format of the manuscript to those required by JAAD International. After using this tool/service, the author(s) reviewed and edited the content as needed and take(s) full responsibility for the content of the published article.

## Conflicts of interest

None disclosed.
